# Design, Formulation and Physicochemical Evaluation of Dimenhydrinate Orally Disintegrating Tablets

**DOI:** 10.22086/gmj.v0i0.936

**Published:** 2018-05-27

**Authors:** Abolfazl Aslani, Alireza Ghasemi, Shekofeh Karbasizadeh Esfahani

**Affiliations:** ^1^Department of Pharmaceutics, School of Pharmacy and Pharmaceutical Sciences, Isfahan University of Medical Sciences, Isfahan, Iran

**Keywords:** Tablets, Dimenhydrinate, Motion Sickness, Meniere’s Disease

## Abstract

**Background:**

Design, formulation and physicochemical evaluation of dimenhydrinate 25 mg oral tablets that disintegrate in oral cavity in a proper time. This product is easy to use for babies, geriatrics and people who have difficulty in swallowing.

**Materials and Methods:**

31 formulations were designed in 3 categories via Design-Expert software version 7. Group 1 consist of super-disintegrating bases, group 2 consist of effervescent bases and group 3 consist of super-disintegrating and effervescent bases together. Proposed by DesignExpert software, the optimum formulations were selected in each category and the tablets were produced by direct compression method. Tablets evaluated by friability, thickness, hardness, weight variation, drug content, content uniformity, disintegration time, wetting time, dissolution and moisture uptake tests.

**Results:**

The angle of repose and compressibility index of formulations were in the range of 24.65-29.08 and 5.02-9.01 % respectively. Thickness, hardness, wetting time, friability and content uniformity of formulations were in the range of 3.36-3.84 mm, 33.25-38.03 N, 19-37 seconds, 0.31-0.42 % and 96.44-99.02 % respectively. Disintegration time of the groups 1, 2 and 3 were in the range of 16-70, 47-72 and 12-35 seconds respectively.

**Conclusion:**

Mixture of powders and orally dispersible tablets passed all tests. The results showed that formulations containing both of super-disintegrants and effervescent bases had better disintegration time compare to other formulations.

## Introduction

Oral drug delivery systems have wide admission up to 50-60% of total solid dosage forms. Solid dosage forms are favorite because of simplicity of administration, exact dosage, self-medication, pain avoidance and significantly the patient compliance [1]. Low disintegration time of tablet results in fast dissolution and speedy absorption which prepare rapid onset of clinical outcome [2].

Pre-gastric absorption of drugs from mouth may be illustration improved oral bioavailability [3]. Geriatric patients may find the administration of the traditional oral dosage forms hard as they regularly require medicines to retain a healthy life. Children may also have problem in absorbency because of their nervous systems and underdeveloped muscular. The problem of devouring tablets is also apparent in journey patients who may not have ready access to water.

Above-mentioned problem can be resolved by means of orally disintegrating tablets (ODTs) [4]. Over a decade, the request for expansion of ODTs has enormously improved as it has important impact on the patient compliance. ODTs suggest an advantage for patients who have problem in consuming.

It has been reported that dysphagia is usual among all age groups and particular with pediatric, geriatric population along with institutionalized patients and patients with vomiting, nausea and motion sickness complications. ODTs with good taste and flavor improve the tolerability of bitter drugs by different groups of population. ODTs are also named as orodispersible tablets, quick disintegrating tablets, fast disintegrating tablets, mouth dissolving tablets, fast dissolving tablets, rapid dissolving tablets and porous tablets [5].

However, of all the above idioms, United States pharmacopoeia (USP) confirmed these dosage forms as ODTs. Currently, European Pharmacopoeia has used the idiom orodispersible tablet for tablets that disperses readily and within 3 min in mouth before swallowing. United States Food and Drug Administration (US FDA) has been described ODTs by “A solid dosage form containing medicinal substance or active ingredient which disintegrates rapidly usually within a matter of seconds when placed upon the tongue”. The disintegration time for ODTs commonly confines from several seconds to about a minute [6, 7]. Dimenhydrinate is a salt of diphenhydramine and 8-chlorotheophyline. Dimenhydrinate affect the body by antihistaminic and anticholinergic properties. Diphenhydramine has antagonistic influence on H1 receptor for inhibition and treating of nausea, vomiting, motion sickness and Meniere's disease. Meniere's disease is a persistent sickness that affects a considerable number of patients every year in the world. The disease is distinguished by periodic episodes of vertigo lasting from minutes to hours, with undulating intuitive hearing loss, tinnitus, and aural pressure. Although there is no cure at the moment, more than 85% of patients involve with Meniere's disease are relieved by medicinal treatment like dimenhydrinate. Drowsiness of diphenhydramine is neutralized by 8-chlorotheophylline. Dimenhydrinate is applied as an over-the-counter (OTC) and self-medication drug. Nausea and vomiting in order to motivating chemoreceptor trigger zone (CTZ) is treated by dimenhydrinate 5–12.5 mg for pediatric patients and 50-100 mg for adults at least 30 min before the journey every 6 hours are suitable dosage of dimenhydrinate. This dose may be repeated every 4h if required, but a total daily dose of 300 mg should not be exceeded [8, 9]. The effect of debilitating central nervous system and cause sedation, drowsiness, confusion and lack of coordination. Antimuscarinic properties, such as atropine can cause side effects, including dry mouth, decreased secretion of the respiratory tract, urinary retention, decreased gastrointestinal motility and constipation are dimenhydrinate side effects [10]. Dimenhydrinate is instable in gastrointestinal pH, by oral administration the absorption of the drug is variable and undergoes widespread first pass metabolism, after oral administration, bioavailability is 46% [9]. Dimenhydrinate's dosage forms in the world pharmaceutical market has capsules 50 mg, chewable tablets 50 mg, syrups 5 mg/2 ml and 3 mg /ml, injectable 50 mg/ml and Suppositories 25, 50 and 100 mg [11]. The goal of this study was to design, formulate and evaluate the physiochemical properties of dimenhydrinate 25 mg ODTs in order to decrease disintegration time in oral cavity as well as providing patient convenience, particularly for people with swallowing difficulties. By using super-disintegrants and effervescent bases improve water uptake with minimum wetting time and accordingly reduce the disintegration time.

## Materials and Methods

### 1. Materials

The materials used in the formulations with their manufacture/sources of ingredients were as follows: Dimenhydrinate was obtained from Tehran Daru Pharmaceutical Company (Tehran, Iran). Sodium starch glycolate (SSG), cross carmellose sodium (CCS), coss povidone (CP), microcrystalline cellulose and also flavoring agents such as cherry and tutti-frutti were provided by Farabi Pharmaceutical Company (Isfahan, Iran). Citric acid anhydrous,

Na bicarbonate, menthol, mannitol, Mg stearate and polyethylene glycol 6000 (PEG 6000) were purchased from Merck (Germany) and sucralose was supplied by Kamvar company (Isfahan, Iran).

### 2. Spectrophotometric Analysis

#### 2.1. Determination of Dimenhydrinate λ_max_ in Phosphate Buffer pH 6.8 and Purified Water

For determination of dimenhydrinate λ_max_ in phosphate buffer and purified water, absorbancies of standard solution were measured in the wavelengths of 200, 210, 220, 230, 240, 250 and 400 nm using 1cm quartz cell.

#### 2.2. Determination of Dimenhydrinate Standard Curve in Phosphate Buffer pH 6.8

10 mg of dimenhydrinate powder transfer into 250 ml volumetric flask and diluted by phosphate buffer pH 6.8. By transferring 0.31, 0.62, 1.25, 2.5, 5 and 10 ml from this solution into a series of 25 ml of volumetric flasks and diluted by

phosphate buffer pH 6.8 to prepare the concentrations of 0.5, 1, 2, 4, 8 and 16 μg/ml, respectively. Absorbancies of these solutions measured at λ_max_ in phosphate buffer pH 6.8 and this method was taken 3 times per day for 3 following days.

#### 2.3. Determination of Dimenhydrinate Standard Curve in Purified water

10 mg of dimenhydrinate powder transfer into 250 ml volumetric flask and diluted by purified water. By transferring 0.31, 0.62, 1.25, 2.5, 5 and 10 ml from this solution into a series of 25 ml of volumetric flasks and diluted by purified water to prepare the concentrations of 0.5, 1, 2, 4, 8 and 16 μg/ml, respectively. Absorbance of all solutions measured at λ_max_ in purified water and this method was taken 3 times per day for 3 following days.

### 3. Evaluation of Powder Mixture

The angle of repose, compressibility index and Hausner’s ratio are the main flowability properties of mixed powders.

#### 3.1. Angle of Repose (θ)

Angle of repose is the internal angle between the surface of the mass of blend and the horizontal surface. By passing the blend through a funnel permanent to a burette foundation at a special height (4 cm) the angle of repose was known. The radius and height of the mass was measured. Angle of repose was calculated by using the formula [12]:

θ = tan ^-1^ (h / r)

h = Height of the mass

r = Radius of the mass

#### 3.2. Bulk Density (ρb) and Tapped Density (ρt)

Both ρb and ρt density were determined. A proper amount of powder from each formulation, formerly lightly shaken to separate agglomerates formed, was presented into a 10 ml measuring cylinder. As soon as initial volume was observed, the cylinder was permissible to fall under its own weight on to a hard surface from a height of 2.5 cm at 2 seconds period. The tapping was followed until no major change in volume was not ed. *ρb* and *ρt* were calculated by using following formula [16]:

ρb = weight of the powder / volume of the packing

ρt = weight of the powder / tapped volume of the packing

#### 3.3. Compressibility Index

It is a plain test to appraise ρt and ρb of a powder and the level at which it packed down. The compressibility index formula is as [13]:

Compressibility index (%) = [(*ρt–ρb*) /*ρt*] * 100

#### 3.4. Hausner’s Ratio

It shows the flow physical characteristics of the powder. The ratio between tapped density to the bulk density of the powders is named Hausner’s ratio [13]:

Hausner’s ratio = *ρ*Tapped / *ρ*Bulk

### 4. Experimental Design

The product variables that affects product quality was investigated by Box-Behnken in Design-Expert version7 (DX7) software. Using the selected independent variable, a Box-Behnken design study was planned and the effect on dependent variable was measured. Based on relationship between dependent and independent variables, optimum formula was determined. The ODTs were designed in 3 groups. First group was designed applying super-disintegrating materials with 3 independent and 2 dependent variables.

Factor A was sodium starch glycolate (SSG) in three levels (3, 7.5 and 12 mg), factor B was cross carmellose (CCS) in three levels (3.5, 5.25 and 7 mg) and factor C was cross povidone (CP) in three levels (3, 5.25 and 7.5 mg). Second level was designed applying effervescent materials with 2 independent and 2 dependent variables.

Factor A was citric acid anhydrous in three levels (9, 18 and 27 mg) and factor B was Na bicarbonate in three levels (13, 24 and 35 mg).

The effervescent components and the ratios between them were specified the neutralization of acids and alkali and the admissible amount of each component. Third group was designed applying effervescent and super-disintegrating materials with 2 independent and 2 dependent variables. Factor A was citric acid anhydrous in three levels (12, 24 and 36 mg) and factor B was cross povidone in three levels (4, 7 and 10 mg)

(Tables-1, 2 and 3). The weight of tablets in group 1 and 2 was 150 mg and in group 3 was 200 mg. Ratios between effervescent components were specified according to neutralization of acid and alkali. In this study, designed by DX7 software, 2 dependent variable has been analyzed as answer; disintegration time and friability test of prepared ODTs.

**Table-1:** Formulations Designed by Design-Expert Software with Superdisintegrant Bases

**Formulations**	**SSG** ** (mg)**	**CCS** **(mg)**	**CP** **(mg)**	**Friability** **(%)**	**Disintegration time (sec)**
**F**_1_	7.50	7.00	3.00	0.40	70
**F**_2_	7.50	7.00	7.50	0.36	16
**F**_3_	7.50	3.50	3.00	0.32	75
**F**_4_	12.00	5.25	7.50	0.38	19
**F**_5_	3.00	7.00	5.25	0.41	28
**F**_6_	12.00	5.25	3.00	0.35	72
**F**_7_	7.50	5.25	5.25	0.36	33
**F**_8_	3.00	3.50	5.25	0.32	44
**F**_9_	12.00	7.00	5.00	0.37	28
**F**_10_	3.00	5.25	3.00	0.33	68
**F**_11_	3.00	5.25	7.50	0.42	22
**F**_12_	7.50	3.50	7.50	0.41	24
**F**_13_	12.00	3.50	5.25	0.37	35

**SSG:** Sodium starch glycolate; **CCS:** Croscarmellose sodium; **CP:** Crospovidone

**Table-2:** Formulations Designed by Design-Expert Software with Effervescent Bases

**Formulations**	**Citric acid** **(mg)**	**Na bicarbonate (mg)**	**Friability** **(%)**	**Disintegration time (sec)**
**F**_14_	27.00	24.00	0.40	49
**F**_15_	9.00	24.00	0.31	72
**F**_16_	18.00	24.00	0.36	61
**F**_17_	9.00	35.00	0.32	69
**F**_18_	18.00	13.00	0.33	77
**F**_19_	18.00	35.00	0.33	58
**F**_20_	9.00	13.00	0.31	75
**F**_21_	27.00	13.00	0.37	64
**F**_22_	27.00	35.00	0.42	47

**Table-3:** Formulations Designed by Design-Expert Software with Superdisintegrant and Effervescent Bases

**Formulations**	**Citric acid** **(mg)**	**Na bicarbonate** **(mg)**	**CP** **(mg)**	**Friability** **(%)**	**Disintegration time (sec)**
**F**_23_	24.00	48	4.00	0.36	32
**F**_24_	24.00	48	7.00	0.37	21
**F**_25_	36.00	48	4.00	0.39	25
**F**_26_	12.00	48	10.00	0.38	22
**F**_27_	12.00	48	7.00	0.36	28
**F**_28_	36.00	48	7.00	0.39	21
**F**_29_	24.00	48	10.00	0.40	18
**F**_30_	36.00	48	10.00	0.41	12
**F**_31_	12.00	48	4.00	0.36	35

**CP:** Crospovidone

### 5. Preparation of Tablets

All ingredients from every formulation were weighed separately. Manitol, avicel, super-disintegrating or effervescent materials were mixed for 5 minutes, flavoring agent and sucralose were added afterwards to the blend and mixed. Finally, the lubricant agent (Mg stearate or PEG 6000) was added and then mixed for 5 minutes again. The tablets were compressed and the weight of tablets were determined as 150 mg and 200 mg (Tables-4 and 5 and 6). Round flat-shaped tablets were produced using die and punch 8 mm (Kilian & Co, Germany).

**Table-4:** Ingredients for Dimenhydrinate ODTs with Superdisintegrant Bases

**Ingredients** **(mg)**	**Formulations**												
	**F**_1_	**F**_2_	**F**_3_	**F**_4_	**F**_5_	**F**_6_	**F**_7_	**F**_8_	**F**_9_	**F**_10_	**F**_11_	**F**_12_	**F**_13_
**Dimenhyrinate**	25	25	25	25	25	25	25	25	25	25	25	25	25
**SSG**	3	3	3	3	7.5	7.5	7.5	7.5	7.5	12	12	12	12
**CCS**	7	3.5	5.25	5.25	7	7	3.5	5.25	3.5	5.25	5.25	7	3.5
**CP**	5.25	5.25	3	7.5	3	7.5	3	5.25	7.5	7.5	3	5.25	5.25
**MCC**	30	30	30	30	30	30	30	30	30	30	30	30	30
**Sucralose**	5	5	5	5	5	5	5	5	5	5	5	5	5
**Mannitol**	72.75	76.25	76.75	72.25	70.5	66	74	70	69.5	63.25	67.75	63.75	65
**Mg stearate**	2	2	2	2	2	2	2	2	2	2	2	2	2
**Total weight**	150	150	150	150	150	150	150	150	150	150	150	150	150

**SSG:** Sodium starch glycolate; **CCS:** Croscarmellose sodium; **CP:** Crospovidone; **MCC:** Microcrystalline cellulose

**Table-5:** Ingredients for Dimenhydrinate ODTs with Effervescent Bases

**Ingredients(mg)**	**Formulations**												
	**F**_14_	**F**_15_	**F**_16_	**F**_17_	**F**_18_	**F**_19_	**F**_20_	**F**_21_	**F**_22_
**Dimenhydrinate**	25	25	25	25	25	25	25	25	25
**Citric acid**	9	9	9	18	18	18	27	27	27
**Na bicarbonate**	13	24	35	13	24	35	13	24	35
**Sucralose**	5	5	5	5	5	5	5	5	5
**PEG**_6000_	4	4	4	4	4	4	4	4	4
**Mannitol**	94	83	72	85	74	63	76	65	54
**Total weight**	150	150	150	150	150	150	150	150	150

**Table-6:** Ingredients for Dimenhydrinate ODTs with Superdisintegrant and Effervescent Bases

**Ingredients** **(mg)**	**Formulations**												
	**F**_23_	**F**_24_	**F**_25_	**F**_26_	**F**_27_	**F**_28_	**F**_29_	**F**_30_	**F**_31_
**Dimenhydrinate**	25	25	25	25	25	25	25	25	25
**Citric acid**	12	12	12	24	24	24	36	36	36
**Na bicarbonate**	36	36	36	36	36	36	36	36	36
**CP**	4	7	10	4	7	10	4	7	10
**Sucralose**	5	5	5	5	5	5	5	5	5
**MCC**	40	40	40	40	40	40	40	40	40
**PEG**_6000_	4.6	4.6	4.6	4.6	4.6	4.6	4.6	4.6	4.6
**Mannitol**	71.4	68.4	65.4	59.4	56.4	53.4	47.4	44.4	41.4
**Mg stearate**	2	2	2	2	2	2	2	2	2
**Total weight**	200	200	200	200	200	200	200	200	200

**CP:** Crospovidone; **MCC:** Microcrystalline cellulose

### 6. Physicochemical Evaluation of the Prepared Tablets

#### 6.1. Weight Variation

Randomly, 20 tablets were chosen after compression and the average weight was determined. None of the tablets deviated from the mean weight by more than ±7.5 % [13, 14].

#### 6.2. Friability Test

Friability test was accomplished to determine the effects of shock and friction. 10 tablets were weighed and put in the friabilator machine (Erweka, TAP, Germany) and regulated on the speed of 25 rpm for 4 minutes. The separated particles of the tablets were removed cautiously and tablets were reweighed. Compressed tab¬lets should not decrease more than 1% of weight. Friability percentage was calculated by following equation [15, 16].

*Friability(*%)=(primary weight of the tablets –terminal weight of the tablets)/(primary weight of the tablets)×100

#### 6.3. Thickness Test

This test was determined for 20 tablets of each formulation using a Vernier caliper and the mean thickness was determined in mm. The variation limit of thickness should be controlled within a ±5% of a standard [12].

#### 6.4. Hardness Test

In this study, ten tablets were chosen randomly and individually located in a hardness tester (Erweka, 24-TB, Germany) and then the hardness of tablets described in Newton. Hardness in the ODTs was usually less than conventional tablets [16].

#### 6.5. Assessment

Twenty ODTs were weighed and powdered. The powder equivalent to 25 mg dimenhydrinate was weighed accurately and dissolved in 25 ml of phosphate buffer pH 6.8.

The solution was shake excellently. By filtration through Whatmann No.41 filter paper the undissolved materials was eliminated.

Then the serial dilutions were prepared. The absorbance of diluted solutions measured at λ_max_ in phosphate buffer pH 6.8. The concentration of the drug was calculated from the standard curve of the dimenhydrinate in phosphate buffer pH 6.8 [17].

#### 6.6. Content Uniformity

Ten tablets of each formulation were weighed and powdered. Aliquot of this powder containing 25mg of dimenhydrinate was accurately weighed, added 50 ml of phosphate buffer pH 6.8 and shaken for 15 minutes. Final volume was regulated to 100 ml with phosphate buffer pH 6.8 and filtered (Whatman No.1 filter paper).

From this solution, 10 ml was diluted to 100 ml.

2 ml of this solution diluted to 10 ml with phosphate buffer pH 6.8 to made final solution. Absorbance of this solution was noted at λ_max_ in phosphate buffer pH 6.8 using UV/Vis spectrophotometer against a blank and the results was compared from a calibration curve prepared with standard dimenhydrinate in the similar medium [18].

#### 6.7. In-Vitro Disintegration Time

The test was done on six tablets using the fixed basket containing six cylindrical glass tubes, stainless steel basket with certain mesh is the bottom of each tube. Six tablets of every formulation were used to calculate disintegration time. Purified water was disintegration medium and temperature was maintained 37±2°C. Disintegration time of six tablets was determined [19].

#### 6.8. Wetting Time

A part of twice-folded tissue paper was put in a small petri dish (internal diameter of 5.5 cm) containing 6 ml of purified water. A tablet was located on the paper and the time required for ending wetting time was computed [20].

#### 6.9. In-Vitro Dissolution Studies

Dissolution testing of dimenhydrinate ODTs was done with paddle method in USP dissolution apparatus at 50 rpm and temperature 37±0.5°C in purified water [13]. 5 ml sample was eliminated and replaced by purified water at times of 1, 2, 3, 4, 5 and 6 minutes to determine the concentration by UV spectroscopy method at λ_max_ in purified water.

### 7. Taste Evaluation of the Prepared Tablets

To assessment the taste, by Latin-square method, the panel tests were done. At first, several flavoring agents such as, menthol, tutti-frutti, cherry and without flavor were prepared for formulations but the amount of excipients, sweeteners and active ingredient were fixed. 20 healthy volunteers were selected and separated into four categories: group one was given cherry and menthol (A), tutti-frutti and menthol (B), menthol (C) and without flavoring agents (D). The group two: B, C, D and A, group three: C, D, A and B and the group four was the D, A, B and C. Then, the volunteers were inquired to score each of the formulation from 1 to 5 (1: bad, 2: poor, 3: average, 4: good, 5: very good taste) [21].

### 8. Moisture Uptake Study

ODTs usually have high concentration of hydrophilic excipients with the minimum possible hardness which together contributes to their increased capacity to moisture uptake. Moisture uptake studies for ODTs should be steered into the stability of the formulation; thus, moisture uptake study is a significant phase in the case of ODTs. Moisture uptake studies was done by weight design. Ten tablets were put in the desiccators over calcium chloride at 37°C for 24 hours to certify that all tablets were dried completely. The tablets were weighed and exposed to 75% RH at room temperature for 14 days. The required humidity can be attained by keeping saturated sodium chloride solution at the underneath of the desiccators for 3 days. The tablets were weighed again and the percent increase in weight was recorded [22, 23].

## Results

The λ_max_ of dimenhydrinate solution in phosphate buffer pH 6.8 was 279 nm. The standard curve of dimenhydrinate in phosphate buffer pH 6.8 was determined spectrophotometrically by curve equation y= 0.0443x + 0.003 and the regression was R² = 0.999. The λ_max_ of dimenhydrinate solution in purified water was 276 nm.

The standard curve of dimenhydrinate in purified water was determined spectrophotometrically by curve equation

y= 0.0322x + 0.005 and the regression was

R² =0.999.Designed formulations by Design-Expert software elucidated in Table-1, 2 and 3. Design-Expert software proposed one optimum formulation for each group (Table-7). The formulation mixed powders was characterized via different tests such as bulk density, tapped density, angle of repose, Husner’s ratio and compressibility index (Table-8). Selected formulations of groups 1(OS), 2(OE) and 3(OSE) were analyzed by different tests such as thickness, hardness, weight variation, friability, disintegration time, assay, content uniformity, wetting time and water content (Table-9). Analyzed tests for optimum mixed powder and ODTs were done (Tables-8 and 9. Weight of 20 tablets in groups 1 and 2 were in range of 146 mg to 152 mg and 147 mg to 151 mg, respectively; and in group 3 was in range of 195 mg to 203 mg. Friability, thickness and hardness of optimum formulations of groups 1, 2 and 3 were 0.31-0.42%, 3.36-3.84 mm, 33.25-38.03 N respectively.

**Table-7:** Optimum Formulations That Proposed by Design-Expert Software

**Formulations**	CP (mg)	CCS (mg)	SSG (mg)	SB (mg)	CA (mg)	Friability (%)		Disintegration time (sec)	
						E	O	E	O
**OS**	7.09	6.74	4.84			0.39	0.38	16.97	17.36
**OE**	-	-	-	34.70	26.52	0.39	0.39	47.90	46.21
**OSE**	9.97	-	-	36	30.96	0.38	0.37	11.89	12.35

**SSG:** Sodium starch glycolate; **CCS:** Croscarmellose sodium; **CP:** Crospovidone; **SB:** Na bicarbonate; **CA:** Citric acid; **E:** Estimated; **O:** Obtained; **OS:** Optimum Superdisintegrant formulation, **OE:** Optimum Effervescent formulation; **OSE:** Optimum Superdisintegrant and Effervescent formulation

**Table-8:** Evaluation of Physicochemical Characteristics of Mixed Powders

**Formulations**	** Physicochemical properties (mean ±SD)**				
	**Bulk density** **(g/cm** ^3^ **)**	**Tapped density(g/cm** ^3^ **)**	**Angle of repose**	**Husner’s ratio**	**Compressibility index (%)**
**F**_1_	0.59±0.03	0.66±0.03	24.67±0.91	1.12±0.02	5.02±0.21
**F**_2_	0.64±0.02	0.65±0.08	26.54±1.16	1.01±0.01	7.34±0.12
**F**_3_	0.61±0.02	0.67±0.04	25.52±0.88	1.10±0.01	6.89±0.18
**F**_4_	0.58±0.03	0.65±0.02	28.36±0.98	1.12±0.04	8.35±0.16
**F**_5_	0.60±0.01	0.68±0.06	25.89±1.01	1.13±0.02	6.05±0.13
**F**_6_	0.63±0.04	0.67±0.01	27.33±0.93	1.06±0.03	7.76±0.23
**F**_7_	0.62±0.07	0.65±0.04	26.96±1.08	1.05±0.06	8.32±0.14
**F**_8_	0.64±0.04	0.66±0.02	25.09±0.90	1.03±0.03	4.97±0.19
**F**_9_	0.62±0.02	0.68±0.07	26.22±1.13	1.10±0.05	5.68±0.10
**F**_10_	0.65±0.03	0.66±0.09	28.98±0.98	1.01±0.02	9.01±0.27
**F**_11_	0.59±0.08	0.65±0.03	26.39±1.09	1.10±0.07	8.87±012
**F**_12_	0.64±0.07	0.68±0.05	27.69±1.12	1.06±0.08	7.56±0.16
**F**_13_	0.62±0.05	0.66±0.07	29.01±1.15	1.06±0.02	5.97±0.20
**F**_14_	0.66±0.04	0.69±0.09	28.69±0.99	1.04±0.04	8.34±0.19
**F**_15_	0.62±0.02	0.66±0.02	26.48±1.08	1.06±0.03	6.64±0.14
**F**_16_	0.63±0.06	0.68±0.04	27.99±0.95	1.08±0.06	5.83±0.15
**F**_17_	0.59±0.08	0.65±0.07	25.47±1.19	1.10±0.04	8.84±0.17
**F**_18_	0.63±0.02	0.68±0.04	26.64±1.14	1.08±0.03	5.76±0.23
**F**_19_	0.61±0.06	0.65±0.07	28.54±0.98	1.06±0.06	5.03±0.12
**F**_20_	0.63±0.03	0.67±0.05	27.08±1.02	1.06±0.02	5.93±0.13
**F**_21_	0.62±0.02	0.66±0.03	28.37±0.93	1.06±0.8	8.91±0.16
**F**_22_	0.58±0.08	0.64±0.04	26.78±0.93	1.10±0.01	7.98±0.18
**F**_23_	0.62±0.04	0.68±0.08	28.39±1.20	1.10±0.05	5.74±0.28
**F**_24_	0.65±0.03	0.67±0.05	28.91±0.92	1.03±0.03	6.99±0.12
**F**_25_	0.62±0.04	0.66±0.07	24.65±1.02	1.06±0.09	7.82±0.08
**F**_26_	0.62±0.05	0.68±0.04	25.63±0.94	1.10±0.02	6.68±0.13
**F**_27_	0.64±0.05	0.69±0.01	24.96±1.08	1.08±0.04	7.14±0.07
**F**_28_	0.63±0.03	0.64±0.06	26.04±1.23	1.02±0.03	8.13±0.21
**F**_29_	0.61±0.02	0.68±0.04	29.08±1.06	1.11±0.06	6.48±0.12
**F**_30_	0.62±0.06	0.67±0.07	27.64±0.96	1.08±0.02	7.56±0.19
**F**_31_	0.60±0.01	0.65±0.02	25.13±1.21	1.08±0.09	6.05±0.19
**OS**	0.59±0.06	0.64±0.09	26.17±1.32	1.08±0.03	6.32±0.41
**OE**	0.61±0.04	0.68±0.06	27.75±1.09	1.11±0.08	8.16±0.36
**OSE**	0.67±0.10	0.69±0.12	26.87±1.18	1.03±0.02	7.93±0.25

**OS:** Optimum Superdisintegrant formulation; **OE:** Optimum Effervescent formulation; **OSE:** Optimum Superdisintegrant and Effervescent formulation

**Table-9:** Physicochemical Properties of the Optimum ODTs Prepared by Direct Compression Method

**Formulations**	**Physicochemical properties (mean ±SD)**						
	Thickness (mm)	Hardness (N)	Weight variation (mg)	Assay (mg)	Content uniformity (%)	Water content (%)	Wetting time(sec)
**OS**	3.36±0.06	38.03±1.97	148.0±1.69	24.86±1.21	98.75±1.25	0.29±0.11	22±0.87
**OE**	3.44±0.08	33.25±2.36	150.9±1.36	25.06±1.53	96.44±1.67	0.45±0.19	37±0.98
**OSE**	3.84±0.16	36.23±1.35	198.86±1.73	24.93±1.12	99.02±1.94	0.36±0.23	19±1.13

**OS:** Optimum Superdisintegrant formulation; **OE:** Optimum Effervescent formulation; **OSE:** Optimum Superdisintegrant and Effervescent formulation

**Figure-1:**
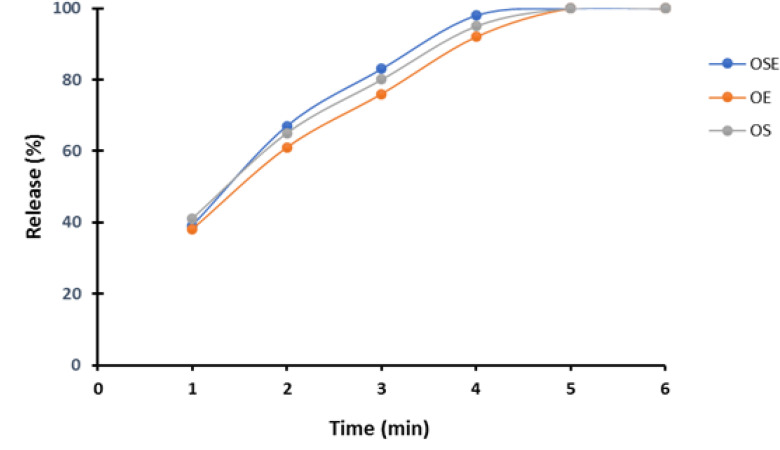
In vitro dissolution test for optimum ODTs in purified water 37±0.5°C

Wetting time, that facilitates faster dispersion in oral cavity, was in range of 19-37 sec. Drug content of optimum formulations was in range of 96.44-99.02 %. The in-vitro disintegration time of groups 1, 2 and 3 were in range of 16-70 seconds, 47-72 seconds and 12-35 seconds, respectively. Results of in-vitro dissolution are shown in Figure-1. Moisture uptake studies showed that results were in range of 0.15-0.55 at 75 % RH. ODTs with combination of tutti-frutti and menthol flavoring has given the best score by volunteers.

## Discussion

Dimenhydrinate is mostly used to treat nausea, vomiting and dizziness caused by motion sickness. Dimenhydrinate has also been found to help in the treatment of ear congestion. Dimenhydrinate’s ODT is useful for peoples with problem in swallowing and don’t access to water. ODTs have pre-gastric absorption and help to by-passing the hepatic first pass effect. Formulations with disintegration time over 60 sec and friability out of range 20% to 40% were ignored by Design-Expert. Standard curve of dimenhydrinate in phosphate buffer pH 6.8 and purified water was plotted by UV spectrophotometry to analyzing assay test, content uniformity and in-vitro dissolution test. By Design-Expert software, all formulations were designed, F_1_-F_13_ formulations (group 1) were designed with different amount of SSG, CCS, and CP, F_14_-F_22_ formulations (group 2) were designed with different amount of citric acid and Na bicarbonate and F_23_-F_32_ formulations (group 3) were designed with different amount of citric acid and cross povidone. By neutralization of acid and alkali, the effervescent components and ratios between them were determined. Tablets with disintegration time over 60 seconds were ignored. Design-Expert program proposed optimum formulation for each group. Formulations with less disintegration time and friability between 0.30-0.40 % were selected. After calculating error percentage, OS (optimum formulation of group 1), OE (optimum formulation of group 2) and OSE (optimum formulation of group 3) were chosen as final formulations for every group.

One of important factor that affect on powder flow is angle of repose. In this study angle of repose was in the range of 24.65 to 29.08. According to USP, all formulations had superfine flow for compression and so tablets were prepared. In other study on zolmitriptan ODTs angle of repose was in the range of 22.32 to 48.42 [12], which confirm our results. The angle of repose under 30, between 31-35, 36-40, 41-45, 46-55, 56-65 and over than 66 has excellent, good, fair, passable, poor, very poor and very, very poor powder flow, respectively [13]. Hausner’s ratio is another important factor that effect on powder flow. In this study hausner’s ratio was in the range of 1.01 to 1.13. According to USP the hausner’s ration between 1-1.11 is excellent, 1.12-1.18 is good, 1.19-1.25 is fair, 1.26-1.34 is passable, 1.35-1.45 is poor, 1.46-1.59 is very poor and over than 1.60 is very, very poor [13]. Results in this study showed that flow of powders were excellent and good.

Hardness of conventional tablets are more than ODTs. In this study hardness of tablets was in the range of 33.25 to 38.03 N. In other studies on ondansetron, metoclopramide and rizatriptan ODTs, hardness was in the range of 20-40 N [24-26], which confirm our results.

Friability in 3 groups was less than 1% and in range of 0.31-0.42%. In other study on piroxicam ODTs friability was in the range of 0.33-0.66%, [27] that confirm our results. Friability and hardness results showed that all tablets had proper mechanical strength.

According to USP, for tablets which their weight are between 130-324 mg, only two tablets can be out of range of ±7.5% of weight (for tablets 150 mg weight ±11.25 and for tablets 200mg weight ±15 mg) [13]. In groups 1 and 2, weight of tablets were in the range of 150 ±11.25 mg; and in group 3 tablets weight were in the range of 200 ±15 mg. Content uniformity test was done to determine the true dosage of each tablet. The range of content uniformity were within 85-115% limitation specified in the USP 38-NF 33 for dimenhydrinate tablets, in this study content uniformity were between 96.44-99.02%, elucidating that the powders were mixed well before compression. All tablets were in the range [24]. The most important test for preparation of ODTs is disintegration time test. Shorter disintegration time is better for admission by patients. In groups 1, 2 and 3, disintegration time was in the range of 16-75 seconds, 47-77 seconds and 12-35 seconds, respectively. The formulations with combination of super-disintegrating with effervescent bases were better than formulations with only super-disintegrating and only effervescent bases. In other studies, disintegration time was in the range of 9-72 seconds [25-27]. The range of wetting time was 19-37 seconds. In other studies wetting time has been reported between 9-75 seconds. The results of other studies, with super-disintegrant bases only, confirm our results [24, 25, 27]. In-vitro dissolution test for 3 optimum formulations has shown that drug release profiles of 3 optimum formulations are similar together and 50% of drugs released in 90 seconds. The tutti-frutti plus menthol flavor gained the best score for drug test by volunteers. Moisturizing uptake studies for 3 optimum formulations was done at 75% RH. Results indicated slight moisture uptake was seen by tablets. According to these results, special packing is needed for our ODTs. The materials with moisture stable features should be used for packing such as aluminum strip pack, aluminum blister or polyethylene sealing on blisters [23].

## Conclusion

Dimenhydrinate has antimuscarinic with antihistaminic and important sedative effects. It is mostly used as an antiemetic drug in the inhibition and treatment of motion sickness. Dimenhydrinate directly prevents the stimulation of definite nerves in the brain and internal ear to suppress nausea, vomiting, dizziness, and vertigo. Dimenhydinate decrease vestibular neuronal stimulation because of angular or linear acceleration motions. This study was helping to design and formulation dimenhydrinate ODTs by effervescent and super-disintegrant bases and mixture of two bases. The results of disintegration time indicated that mixture of two bases (group 3) were better than others. F_2_ in group 1, F_14_ in group 2 and F_3_ in group 3 had lowest disintegration time compare other formulations in each group. OSE formulation had best disintegration time compare to all formulations.

## Conflict of Interests

Authors have no conflict of interests.
